# Telomere regulation in pluripotent stem cells

**DOI:** 10.1007/s13238-014-0028-1

**Published:** 2014-02-22

**Authors:** Yan Huang, Puping Liang, Dan Liu, Junjiu Huang, Zhou Songyang

**Affiliations:** 1 Key Laboratory of Reproductive Medicine of Guangdong Province, the First Affiliated Hospital and Key Laboratory of Gene Engineering of the Ministry of Education, School of Life Sciences, Sun Yat-sen University, Guangzhou, 510275 China; 2 Cell-Based Assay Screening Core, Baylor College of Medicine, One Baylor Plaza, Houston, TX 77030 USA; 3 Dan L. Duncan Cancer Center, Baylor College of Medicine, One Baylor Plaza, Houston, TX 77030 USA; 4 Verna and Marrs Mclean Department of Biochemistry and Molecular Biology, Baylor College of Medicine, One Baylor Plaza, Houston, TX 77030 USA

**Keywords:** telomere, pluripotent stem cells, alternative lengthening of telomeres (ALT), regulators

## Abstract

Pluripotent stem cells (PSCs) have the potential to produce any types of cells from all three basic germ layers and the capacity to self-renew and proliferate indefinitely *in vitro*. The two main types of PSCs, embryonic stem cells (ESCs) and induced pluripotent stem cells (iPSCs), share common features such as colony morphology, high expression of Oct4 and Nanog, and strong alkaline phosphatase activity. In recent years, increasing evidences suggest that telomere length represents another important internal factor in maintaining stem cell pluripotency. Telomere length homeostasis and its structural integrity help to protect chromosome ends from recombination, end fusion, and DNA damage responses, ensuring the divisional ability of mammalian cells. PSCs generally exhibit high telomerase activity to maintain their extremely long and stable telomeres, and emerging data indicate the alternative lengthening of telomeres (ALT) pathway may play an important role in telomere functions too. Such characteristics are likely key to their abilities to differentiate into diverse cell types *in vivo*. In this review, we will focus on the function and regulation of telomeres in ESCs and iPSCs, thereby shedding light on the importance of telomere length to pluripotency and the mechanisms that regulate telomeres in PSCs.

## TELOMERES

Telomeres are hexametric repeats of (TTAGGG)_n_ at the chromosomal ends in mammalian cells. During the process of DNA synthesis and cell division, telomeres are shortened as a result of the incomplete replication of linear chromosomes, which is called “end-replication problem”. Telomere attrition, which can cause chromosome recombination, end-to-end fusion, DNA damage, and genome instability, represents one of the nine hallmarks of aging (Lopez-Otin et al., 2013). Many pieces of evidence, including studies of telomerase null mouse models and diseases with premature aging phenotypes such as dyskeratosis congenita (DC), support the notion that telomere shortening is rate limiting for mammalian lifespan and age-related diseases (Blasco et al., 1997; Herrera et al., 1999; Mason et al., 2005).

Usually, telomere length is maintained by the telomerase, containing the reverse transcriptase TERT and the RNA template TERC, as well as associating proteins such as the ribonucleoprotein dyskerin (DKC1) (Cohen et al., 2007). Telomerase is responsible for the *de novo* telomere extension, to prevent telomere exhaustion after multiple rounds of cell division (Greider and Blackburn, 1985, 1989). In addition, the telomere region is bound by a six-protein complex called shelterin/telosome, containing TRF1, TRF2, TPP1, POT1, TIN2 and RAP1, which is crucial for maintaining the structure and function of telomeres (de Lange, 2005; Liu et al., 2004; Xin et al., 2008).

In most cases, the telomerase is only activated in stem cells, germ cells, certain white blood cells, and cancers. In a significant number of cases, however, telomeres can be maintained without the telomerase, through possible mechanisms such as homologous recombination, which has been termed alternative lengthening of telomeres (ALT). ALT has been found to occur in about 10%–15% cancers and is often characterized by co-localization of telomeres with the promyelocytic leukemia (PML) bodies (known as ALT-associated PML bodies (APBs)), exceedingly heterogeneous telomere length, extra-chromosomal DNA circles, and high frequencies of telomere sister chromatid exchange (T-SCE) (Cesare and Reddel, 2010; Chung et al., 2012). ALT tends to occur in tumors such as osteosarcoma and soft tissue sarcomas derived from mesenchymal or neuroepithelial origin (Cesare and Reddel, 2010; Henson et al., 2005; Scheel et al., 2001). Interestingly, it was found that ALT and telomerase pathway could coexist in human cells under certain circumstances (Cerone et al., 2001). By using a telomere-tagged transgenic mouse strain, ALT was recently found to exist in normal mouse somatic cells, but not in the germline (Neumann et al., 2013). Furthermore, it was found that during the early embryo cleavage stage, telomeres are also lengthened by an ALT-like mechanism (Liu et al., 2007).

Increasing evidences indicate that telomeres are tightly linked to epigenetic regulation. Many heterochromatin features can be found in mammalian telomeric or subtelomeric domains, such as trimethylation of H3K9 and H4K20 (Garcia-Cao et al., 2004; Gonzalo et al., 2005), HP1 enrichment (Lachner et al., 2001), low levels of acetylated H3 and H4 (Benetti et al., 2007a), and DNA hypermethylation in subtelomeric region (Gonzalo et al., 2006). These “silenced” features in the nucleosome help to maintain a compressed chromatin structure and telomere length homeostasis.

## PLURIPOTENT STEM CELLS

Pluripotent stem cells, including the well-studied ESCs and emerging iPSCs, promise great potential applications in the medical and drug field. ESCs were first isolated from the mouse inner cell mass (ICM) of blastocysts in 1981 (Evans and Kaufman, 1981; Martin, 1981). In recent years, ESCs can also be derived from somatic cell nuclear transfer embryos (ntESCs), parthenogenetic embryos (pESCs), and androgenetic embryos (aESCs). In 2006, the Yamanaka group successfully obtained induced pluripotent stem cells (iPSCs) by introducing four transcriptional factors into mouse as well as human somatic cells (Takahashi et al., 2007; Takahashi and Yamanaka, 2006). More detailed studies have found that gene expression patterns, epigenetic states, and telomere length status appeared to have been reversed in this reprogramming process (Buganim et al., 2012; Marion et al., 2009; Papp and Plath, 2013). iPSCs resemble ESCs in multiple molecular markers as well as in producing all-iPSC mice by tetraploid complementation technique (Kang et al., 2009; Maherali et al., 2007; Mikkelsen et al., 2008; Takahashi and Yamanaka, 2006; Zhao et al., 2009).

How PSCs maintain their ability for self-renewal and pluripotency is a fundamental issue in cell biology. Studies in recent years have pointed to epigenetic mechanisms that could control the difference between PSCs and somatic cells. Compared with differentiated somatic cells, ESCs have unique features: they have a more “open” conformation of chromatin structure, including the telomeric region. The repressive histone modifications are less prevailing in the ESC genome, compared to those in differentiated cells (Hawkins et al., 2010; Wen et al., 2009). Many transcription factors that control cell fate determination are epigenetically marked by either active (such as methylated H3K4) or repressive (like methylated H3K27) histone modifications. These bivalent chromatin states provide the plasticity for maintaining ESC pluripotency and regulating the expression level of lineage-specific genes during differentiation (Bernstein et al., 2006). For iPSCs, the epigenetic status of successfully induced cells is highly similar to the ESCs, including changes in histone modifications and DNA methylation at the gene loci that are required for the maintenance of pluripotency and lineage specification, as well as efficient activation of the telomerase and elongation of telomeres (Marion et al., 2009; Takahashi et al., 2007; Takahashi and Yamanaka, 2006).

In addition, ESCs may also have evolved more stringent mechanisms to protect genome integrity compared to differentiated cells. For example, ESCs harbor much lower mutation and recombination rate than somatic cells (Cervantes et al., 2002). Moreover, ESCs exhibit hypersensitivity to DNA damage, efficient DNA repair mechanisms and high proficiency in antioxidant defense, which also help to maintain their genome stability (Giachino et al., 2013). The long and stable telomeres often found in ESCs may be an additional mechanism to protect chromosomal integrity (Huang et al., 2011). The telomeres in PSCs, which can be regulated in a telomerase-dependent or independent (e.g. ALT) fashion, are crucial for PSC biology, which will be discussed in the following sections.

## TELOMERES IN PLURIPOTENT STEM CELLS

While the telomere in somatic cells is usually composed of heterochromatin, the genome-wide chromatin structure is relatively open in PSCs. How is the status of telomeric chromatin in PSCs? Recent studies have shown that PSCs contain relatively “open” telomeric chromatin that would switch to become more repressive during differentiation (Azuara et al., 2006; Bernstein et al., 2006; Meshorer et al., 2006). Therefore, the telomeric chromatin of PSCs is likely in a unique and dynamic state that can undergo remodeling during differentiation (Marion et al., 2009; Wong et al., 2009).

One of the common characteristics of ESCs and iPSCs is the expression of transcription factors Oct4, Sox2, and Nanog, as well as constitutively high telomerase activity. PSCs can use the telomerase pathway to elongate their telomeres (Blasco, 2007; Hiyama and Hiyama, 2007). The *in vivo* study of adult stem cells indicated that adequate telomere length is a prerequisite for the functionality of stem cells (Flores et al., 2008).

**Figure 1:**
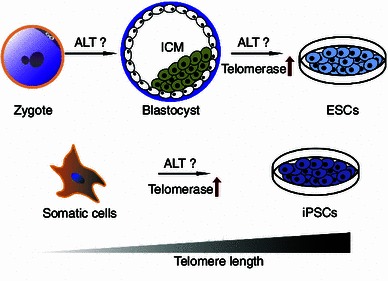
**Telomere length increases during preimplantation embryo development and PSCs derivation process**. At the embryo stage, telomeres are effectively elongated by a possible alternative lengthening of telomeres (ALT) pathway from zygote to blastocyst. However, during early expansion, ESCs obtained from the inner cell mass (ICM) *in vitro* continuously elongate their telomeres to a relatively stable level in a telomerase-dependent way, and ALT pathway could not be excluded in this process. Similarly, both telomerase-dependent and independent mechanism may coexist in the iPSCs reprogramming process. In summary, the PSCs, including ESCs and iPSCs, need to elongate their telomeres during the derivation process to stabilize their genomic DNA and keep their self-renewal and pluripotency status

Dynamic telomere status is not restricted to the aging process, but is also found during embryo development and PSC derivation. At the preimplantation embryo stage, telomeres are effectively elongated by ALT-like mechanism from totipotent zygote to blastocyst and then telomerase is used to maintain telomere length during ontogenesis in adult stem cells (Liu et al., 2007) (Fig. 1). Surprisingly, ICM cells in the blastocyst have different telomere length compared to ESCs—telomeres extend continuously during murine ESC derivation process. There may be multiple non-mutually exclusive mechanisms contributing to ESC telomere elongation: one is that both H3K9me3 and H4K20me3 heterochromatic marks decrease at telomeres; second is high-level expression of TRF1 that facilitates proficient capping of the newly synthesized telomeres. These two factors ensured telomerase dependent elongating of the telomeres. More interestingly, an ALT-like mechanism is also activated during this process to rapidly elongate the telomeres, wherein Zscan4 is involved to maintain genomic stability, (Varela et al., 2011; Zalzman et al., 2010). Similarly, the study of human ESCs demonstrated that telomeres were elongated during early expansion *in vitro* and then reached to a relatively stable level in a telomerase-dependent way (Zeng et al., 2013). For self-renewal and developmental pluripotency, the ESC telomere state is tightly associated with its function. ESCs with short telomeres show reduced pluripotency as revealed by teratoma formation and chimera production *in vivo* (Huang et al., 2011). In Tert^-/-^ ESCs, critically short telomeres induced *de novo* methyltransferase Dnmt3a2/3b downregulation, genome-wide DNA hypomethylation, and altered H3K27me3 enrichment at *Nanog* and *Gata6* promoters as well as at loci distal to telomeres. These epigenetic changes in turn affect the stable differentiation ability of ESCs *in vitro* (Pucci et al., 2013).

During initial iPSC studies, researchers could easily and effectively obtain iPSCs by introducing the four Yamanaka factors into somatic cells. Most of these cell lines showed typical ESC-like colony shape, specific molecular markers such as Oct4, Nanog and Sox2, and positive alkaline phosphatase activity. However, most of these lines could not produce iPSC-mice by tetraploid complementation except chimeric mice, indicating that they were not entirely pluripotent. A recent report found that murine ESCs/iPSCs with short telomeres could not produce ESC/iPSC mice directly and the efficiency of teratoma formation and chimera production decreased significantly (Huang et al., 2011). Also the porcine iPSC studies found that telomere length is related to the efficiency of teratoma formation and ALT mechanism is also found in porcine iPSCs (Ji et al., 2013). Although with some controversy, telomeres can be elongated after somatic cell nuclear transfer (SCNT) process in many species (Jiang et al., 2004; Lanza et al., 2000; Shiels et al., 1999; Sparman et al., 2009). The Blasco group also observed extension of telomeres during iPSC derivation and passage (Marion et al., 2009) (Fig. 1). Recently, the comparison of the differences between the SCNT-mediated and iPSC-based reprogramming of Terc^-/-^ cells found that SCNT is superior to iPSC mediated reprogramming in donor cells with telomere and mitochondria dysfunction, providing an application insight for reprogramming strategy optimization. (Le et al., 2014). Furthermore, ectopic expression of factors, which could protect telomeres such as Zscan4, can increase induction efficiency and pluripotency of iPSCs remarkably (Jiang et al., 2013). These lines of evidence demonstrate that telomere elongation and functional reconstruction is very important for iPSCs to acquire true pluripotency. In fact, the telomeres of iPSCs have been shown to be remodeled to features similar to ESCs by both telomerase-dependent and possible ALT pathway, in both mouse and human (Marion et al., 2009; Wang et al., 2012; Yehezkel et al., 2011) (Fig. 1).

On the other hand, telomere dysfunction can also impact cell metabolism through affecting mitochondrial function. Progressive telomere shortening limits the replication capacity of dividing cells such as stem cells (Cong et al., 2002), and induces p53 to repress PGC-1α and PGC-1β, thereby linking telomeres to mitochondrial biology, oxidative defense, and metabolism (Sahin et al., 2011). One of the core telomeric proteins TIN2 was recently shown to localize to mitochondria, and play an important role in mitochondrial function regulation. TIN2 appears to negatively modulate ATP production by limiting efficient electron transport and decreasing mitochondrial membrane potential (Chen et al., 2012). Whether mutations of TIN2 would affect the pluripotency of PSCs and the efficiency of iPSCs reprogramming in *Tin2*-related dyskeratosis congenital (DC) disease models need further investigation.

Although the mechanism of telomere regulation in the PSCs remains unclear, the following key modulators of ESCs/iPSCs telomere maintenance have been found so far which has expanded our understanding in this field.

### TELOMERASE IN PLURIPOTENT STEM CELLS

The status of the telomerase is crucial for pluripotency of adult stem cells and tissue function. Terc^-/-^ mice with null telomerase activity are viable for only six generations. These mice exhibit premature aging phenotypes such as gray hair, kyphosis, smaller body size and shorter life-span. Conversely, in Tert-overexpressing transgenic mice, telomerase reactivation can reverse tissue degeneration in hair follicles, testes, intestinal crypts and brain in aged telomerase-deficient mice (Flores et al., 2005; Jaskelioff et al., 2011).

Surprisingly, ESCs can be derived from Terc^+/-^ and Terc^-/-^ mice as efficiently as wild type ESCs, indicating that the telomerase *per se* is not required for the establishment of ESC lines. The haplosufficient Terc^+/-^ ESCs had significantly reduced ESC mice production efficiency compared with wild type ESCs. G1 Terc^-/-^ ESCs, derived from first generation Terc^-/-^ mice with normal karyotypes and shorter telomeres, could not produce any ESC mice by 8-cell embryos injecton, even though G1 Terc^-/-^ ESCs could form teratomas as efficiently as Terc^+/-^ and wild type ESCs. The ESCs from G4 Terc^-/-^ mice display aneuploidy and chromosomal abnormalities and could not produce any chimeras (Huang et al., 2011).

Overexpression of telomerase in mouse and human ESCs appeared to improve their self-renewal, colony-formation capacity, resistance to apoptosis and proliferation rate. The Tert-overexpressing human ESCs are able to form teratomas composed of three germ layers *in vivo*, but their ability to differentiate into all primitive and embryonic lineages *in vitro* was suppressed (Armstrong et al., 2005; Yang et al., 2008). In summary, it appears that regulating telomerase activity appropriately in ESCs is critically important for maintaining ESC pluripotency and modulating their differentiation.

Other lines of evidence come from the telomerase-associated diseases. DC is a rare progressive congenital disorder with characteristics of high rates of bone marrow failure, pulmonary fibrosis, oral leukoplakia, nail dystrophy, abnormal skin pigmentation, etc. It is mainly caused by mutations in genes encoding the telomerase or telomerase-associated proteins such as DKC1, TERT, TERC, TCAB1 and RTEL1 (Ballew et al., 2013; Bessler et al., 2010; Walne and Dokal, 2009). These DC patients have very short telomeres in their peripheral blood lymphocytes (Alter et al., 2007). iPSC studies using fibroblasts from DC patients have shed light on the interplay between the telomerase and pluripotency establishment. Reprogrammed iPSCs from Terc destabilized DC patients could overcome the critical limitation in Terc levels to restore telomere maintenance and acquire self-renewal capability (Agarwal et al., 2010). However, iPSCs from different types of DC patients retain the characteristic disease defects, and display telomere maintenance defects that correlate with their corresponding clinical severity (Batista et al., 2011). In most cases, both mutant and wild type iPSCs could upregulate Tert and Terc expression levels compared with parental somatic cells, but mutant iPSCs usually elongated telomeres at a lower rate compared to their wild type counterparts. Defective telomere elongation appears to have a definitive impact on the true pluripotency and differentiation abilities of iPSCs (Winkler et al., 2013).

During either ESCs establishment or iPSCs generation, the mechanism for elongating telomeres seems to be not confined to the telomerase pathway. When Terc-deleted ESCs went through crisis after multiple passages, survived ESCs were found to use ALT pathway to maintain telomeres (Niida et al., 2000). Moreover, although telomerase reactivation is necessary for reprogramming, ALT-dependent mechanism is also involved in reprogramming, in a non-sufficient way (Wang et al., 2012). These results underline the importance of both telomerase-dependent and independent pathways in the regulation of telomeres in PSCs (Fig. 1).

### ALTERNATIVE LENGTHENING OF TELOMERES (ALT) PATHWAY IN PLURIPOTENT STEM CELLS

While 85%–90% of human cancers extend telomeres through the upregulation of telomerase activity, 10%–15% of cancers use the ALT pathway, a process that appears dependent on homologous recombination (Bryan et al., 1997; Shay and Bacchetti, 1997). Usually, the two telomere maintenance mechanisms appear mutually exclusive for most cells. Mutations of *DAXX*, *ATRX* and *H3.3* have also been found to be associated with telomerase-negative human cancer cells that undergo ALT (Schwartzentruber et al., 2012). However, ALT and telomerase pathways may coexist in PSCs, which was supported by the evidence that telomerase reactivation is accompanied by ALT during reprogramming and ALT-related H3.3/ATRX colocalize with telomere within the PML bodies in ESCs (Chang et al., 2013; Wang et al., 2012).

The histone H3 variant H3.3 was originally thought to be commonly associated with active chromatin. However, recent data have revealed that this particular variant can accumulate on silent loci in heterochromatin, indicating its possible dual roles in transcriptional regulation (Goldberg et al., 2010). Interestingly, it was found that H3.3 could localize to the telomeres in mouse ESCs and embryonic germ cells, but not in non-pluripotent cells. H3.3 levels decrease during ESCs differentiation while the heterochromatin repressive markers H4K20me3 and H3K9me3 increase in this process. Meanwhile, depletion of H3.3 could induce dysregulation of telomeres, providing evidence for a role of H3.3 in the regulation of telomere chromatin integrity in ESCs (Wong et al., 2009).

H3.3 can be found in two separate complexes, associating with either histone deposition factor HIRA or histone chaperone DAXX. The HIRA complex can deposit H3.3 at transcribed genes, whereas DAXX and ATRX together with H3.3 assemble at pericentric heterochromatin and telomeres. Strikingly, ATRX and DAXX associate with H3.3 in a HIRA-independent manner in ESCs, which may explain why H3.3 has dual roles at both active and repressed genes (Goldberg et al., 2010). ATRX, short for alpha thalassemia/mental retardation syndrome X-linked gene, belongs to the SWI2/SNF2 family of chromatin remodeling proteins. In cooperation with H3.3 and HP1alpha, ATRX can modulate telomere chromatin status. ATRX knockdown led to increased DNA damage at the telomeres in ESCs, but not in cells such as NIH3T3, indicating a specific role for ATRX in ensuring the integrity of ESC telomere chromatin (Wong et al., 2010b). DAXX or death associated protein is a histone chaperone required for the deposition of H3.3 at telomeres in the context of the ATRX-DAXX complex (Drane et al., 2010; Lewis et al., 2010). Mutations in the ATRX/DAXX complex and histone H3.3 were found to correlate with features of ALT in many cancers (Heaphy et al., 2011; Schwartzentruber et al., 2012). A comprehensive analysis of several ALT cell lines has indicated that loss or mutations of ATRX are hallmarks of ALT cell lines. Additionally, genome instability and altered double-strand break (DSB) repair are also correlated with ALT (Lovejoy et al., 2012). Whether the ATRX/DAXX complex contributes to telomere regulation through the ALT pathway in ESCs needs further investigation.

In ESCs, H3.3, DAXX and ATRX can localize to the promyelocytic leukemia nuclear bodies (PML-NBs) and form ALT-associated PML-NBs (APBs), one of the hallmarks of ALT cells (Chang et al., 2013). Marked by PML and SP100, PML-NBs are subnuclear compartments in eukaryotic cells enriched in regulatory proteins that can modulate multiple cellular processes including gene transcription, tumor suppression, as well as DNA replication and repair (Bernardi and Pandolfi, 2007). The assembly of PML-NBs is correlated with the pluripotent state of mouse ESCs, suggesting the PML-NBs serve as important platforms for the study of telomeric chromatin integrity in ESCs (Chang et al., 2013). Future studies should focus on novel factors in the PML-NBs that regulate ESC telomeres and more details should be demonstrated to unmask the exact mechanism and function of the ALT pathway in PSCs.

### TELOMERE-ASSOCIATED REGULATORS IN PLURIPOTENT STEM CELLS

As mentioned above, the epigenetic state of telomere chromatin may affect telomere length in PSCs. Several reports support the notion that multiple chromatin modulators may participate in the regulation of telomere structure and length. SUV39H1 and SUV39H2, for example, are two histone methyltransferases required for the enrichment of trimethylated H3K9 on constitutive heterochromatin including both centromeres and telomeres. Suv39h1 and Suv39h2 double knockout led to loss of heterochromatic features and abnormal telomere elongation in murine ESCs and porcine embryonic stem like cells, respectively (Dang-Nguyen et al., 2013; Garcia-Cao et al., 2004). Similarly, SUV4-20H1 and SUV4-20H2 are histone methyltransferases responsible for H4K20me3 level maintenance. Simultaneous loss of Suv4-20h1 and Suv4-20h2 resulted in a considerable elongation of telomeres (Benetti et al., 2007b). Recently, it has also been found that abrogation of Suv4-20h decreases the heterochromatic mark H4K20me3 at telomeric regions and facilitates telomere replenish during reprogramming (Marion et al., 2011). Consistently, relatively low density of H3K9me3 and H4K20me3 was observed during reprogramming as well as in ESCs (Marion et al., 2009). This “relaxed” telomeric chromatin state may facilitate the access of telomerase to telomeres in PSCs and thus modulate telomere length.

DNA hypermethylation is another epigenetic feature at subtelomeric regions. The loss of DNA methyltransferases Dnmt1 or Dnmt3a/Dnmt3b in ESCs, which led to hypomethylated subtelomeric chromatin, caused dramatic telomere elongation by homologous recombination, and phenotypes similar to ALT cells, including increased T-SCE, APB formation and telomere heterogeneity. It should be noted that a possible role for the telomerase or other as yet unidentified regulators cannot be excluded in this process (Gonzalo et al., 2006).

Given the importance of telomere maintenance in PSCs, the factors that can regulate telomerase expression, recruitment and activity are also expected to play a significant part in PSCs. For example, the TPP1-POT1 heterodimer in the shelterin/telosome complex binds to single-stranded G-rich telomeric overhangs. TPP1-POT1 association can enhance POT1-DNA affinity and telomerase recruitment. TPP1 RNAi in cancer cells caused DNA damage response at the telomeres and led to telomere dysfunction (Xin et al., 2007). Indeed, abrogation of TPP1 also abolished telomere elongation during reprogramming of mouse embryonic fibroblast (MEF) cells, supporting a role of TPP1 in PSCs (Tejera et al., 2010). Several transcriptional regulators of the telomerase in PSCs have also been demonstrated. Hypoxia inducible factor 1 alpha (HIF1α) was identified as an upstream transactivator of Tert in response to hypoxia in mouse ESCs in a shRNA library screening. HIF1α loss in ESCs is accompanied by both decreased Tert level and shortened telomere length, which in turn can be restored by reintroducing HIFα level in a hypoxic condition (Coussens et al., 2010). Klf4, one of the four Yamanaka factors (Oct4, Sox2, Klf4, c-Myc), was found to specifically and directly activate the Tert promoter in ESCs. Klf4 knockdown in ESCs led to reduced Tert expression and ESC differentiation, whereas overexpression of Tert rescued these phenotypes (Wong et al., 2010a). In cooperation with Klf4, the classical multifunctional Wnt/β-catenin signaling pathway can directly target Tert (Hoffmeyer et al., 2012). These data provide a direct link between PSC pluripotency and telomerase activity regulation.

In addition, novel factors have been found to take part in telomere regulation in PSCs as well. For example, zinc finger and SCAN domain containing 4 (*Zscan4*) is a gene cluster that contains six members, and expressed specifically in late 2-cell embryos and ESCs (Falco et al., 2007). It was found that Zscan4 could elongate telomeres in ESCs by homologous recombination and form foci on telomeres. Thus Zscan4 facilitates and maintains genome stability in ESCs in a telomerase-independent manner (Zalzman et al., 2010). Further studies in reprogramming demonstrate a consistent role of Zscan4 in protecting genome stability of PSCs, and Zscan4 overexpression could rejuvenate the developmental potency of PSCs in long-term cell culture (Amano et al., 2013; Jiang et al., 2013). The precise molecular functions of Zscan4 in the possible ALT pathway in PSCs are still unclear. The investigation of Zscan4 and other novel factors should greatly facilitate our understanding of PSC telomere maintenance mechanism.

## PROSPECT

Multiple links between telomere biology and stem cell pluripotency have been established. Interestingly, the longest telomeres appear to exist in adult stem cells where they become shortened as stem cell function declines. Additionally, higher expression of the shelterin/telosome complex component TRF1 correlates with higher pluripotency of stem cells. Consequently, long telomeres and high TRF1 level have been proposed as stem cell markers (Flores et al., 2008; Huang et al., 2011; Schneider et al., 2013). Indeed, telomere status may serve as a benchmark to evaluate the quality of ESCs and iPSCs, which should have important implications in the application of PSCs in regenerative medicine (Fig. 1). Compared with somatic cells, PSCs have unique combinations of telomere-associated proteins. Elucidating the function of these factors, particularly their specific roles in regulating PSC telomeres would be a top priority. Finally, further understanding of how and why PSCs maintain their telomeres through both the telomerase and possible ALT pathway promises great insight into the complicated mechanisms that establish and ensure pluripotency of stem cells.

## Abbreviations

ALT: alternative lengthening of telomeres
DC: dyskeratosis congenital
DSB: double-strand break
ESCs: embryonic stem cells
ICM: inner cell mass
iPSCs: induced pluripotent stem cells
MEF: mouse embryonic fibroblast
PML: promyelocytic leukemia
PSCs: pluripotent stem cells
